# Simulation study - handling missing covariates in the context of external validation

**DOI:** 10.1186/1745-6215-12-S1-A62

**Published:** 2011-12-13

**Authors:** Laura J Bonnett, Tony G Marson, Paula R Williamson, Catrin Tudur-Smith

**Affiliations:** 1 Department of Biostatistics, University of Liverpool, Liverpool, L69 3GS, UK; 2 Clinical and Molecular Pharmacology, University of Liverpool, Liverpool, L69 3GS, UK

## Objectives

Before a predictive or prognostic model, often developed using data from clinical trials, can be introduced into general practice, it needs to be externally validated to ensure that it performs satisfactorily in data sets that are fully independent of the development data. Various methods exist to handle covariates with missing data and many of these are regularly employed in the analysis of data from a single trial. We propose that several of these strategies may be adapted to handle covariates with every entry missing in the context of external validation.

## Methods

A simulation study was undertaken to test the suitability of our five proposed strategies: (1) random selection with replacement, (2) hot deck imputation, (3) single imputation via estimation, (4) random selection with replacement multiple times, (5) using only covariates common to both development and validation data sets. Survival times were simulated via the Cox-exponential distribution with a binary censoring indicator variable. Up to two binary, two continuous and two categorical covariates were simulated via binomial, log-normal and multinomial distributions respectively. To assess how the methods perform in general three statistics were calculated across 1000 bootstrap samples: (i) estimated regression coefficients from the model fit to the validation set, (ii) associated standard deviations, (iii) mean square errors of the parameter estimates from the development and validation sets.

## Results

Preliminary results suggest that random selection with replacement multiple times was the most consistent method; the mean difference between the actual regression coefficients from the development set and those estimated from the validation set was only 0.02 whereas it was 0.10 for random selection with replacement, 0.09 for imputation via estimation and 0.05 for hot-deck imputation. Standard deviations were fairly constant across methods (1) to (4). Results for method (5) are to follow together with mean square errors for all five methods.

## Conclusion

Random selection with replacement multiple times may offer a solution to externally validating a predictive or prognostic model when at least one covariate is missing from the validation data set. The simulation study described is an over-simplification of reality so leads to more favourable results than can be expected in everyday applications. Similarly it does not consider associations between variables. Further work is required to determine how the methods perform in alternative settings and also in real life.

